# Bibliometric analysis of China’s contribution to the knowledge system of cerebrovascular intervention

**DOI:** 10.1186/s41016-021-00264-y

**Published:** 2021-12-19

**Authors:** Hongyu Ma, He Li, Peng Liu, Pei Liu, Xiaoxi Zhang, Yongxin Zhang, Zifu Li, Rui Zhao, Bo Hong, Jianmin Liu, Pengfei Yang

**Affiliations:** Neurovascular Center, Changhai Hospital, Naval Medical University, Shanghai, China

**Keywords:** Bibliometrics, Cerebrovascular disorders, Endovascular procedures, Interventional radiology, Stroke

## Abstract

**Background:**

Cerebrovascular disease has become the leading cause of death in China. The purpose of this article is to analyze China’s contribution to the interventional treatment of cerebrovascular diseases.

**Methods:**

Bibliometric analysis was used for evaluating the quantity, quality, research hotspots, and cooperation network of publications regarding interventional treatment of cerebrovascular diseases from China. These articles were searched from the database of Web of Science Core Collection. The authors, publication years, citation times, regions, and source journals of retrieved articles were recorded. Network analysis and visualization were performed on Citespace5.6.

**Results:**

From 1991 to 2019, a total of 5052 articles regarding cerebrovascular intervention were contributed by Chinese researchers. The number of publications from China grew fastest annually in the latest 5 years among countries. These publications were cited 61,216 times, with 12.12 average citations per item. The h-index was 82. Affiliated hospitals of Capital Medical University contributed most articles. Cerebral ischemia and intracranial aneurysm were the most popular keywords over the three decades. The timeline view of keywords indicated that cerebral ischemia always was a hot spot. Stent techniques were the main treatment tools and still had a strong developing trend. Neural regeneration and neuroprotection were the hot topics of basic researches related to cerebrovascular intervention.

**Conclusions:**

The number of researches grows rapidly in China over the decades, but the quality still needs further improvement. The increasing contributions of Chinese researchers to the global knowledge system of cerebrovascular intervention are promising.

## Background

Cerebrovascular diseases (CVDs), consisted of conditions that affect cerebral blood supply, impose a global heavy burden on health [1]. Disparities in the incidence of CVDs are growing, with a decrease in high-income countries and an increase in low-income and middle-income countries (LMICs) such as China [2]. According to the Global Burden of Disease Study, CVDs are the leading cause of death in China [3]. Hopefully, over the past three decades, development in cerebrovascular intervention techniques has provided a new treatment strategy for CVDs [4]. Interventional surgery now has become the most common therapeutic approach for cerebrovascular disorders in China [5]. The practice of this technique has brought meaningful benefits to many Chinese patients [6]. But China’s contribution to the knowledge system of cerebrovascular intervention has not been well evaluated.

For the first time, we made the bibliometric analysis to evaluate the contributions of China’s researches to the cerebrovascular intervention knowledge system and provide the visualization of research development with the use of Citespace 5.6 [7]. The purpose of this analysis is to draw experience from the past, predict the trends in development, and get inspiration for future research [8, 9]

## Methods

### Literature retrieve

To select Chinese cerebrovascular intervention research with global influence, we chose Science Citation Index Expanded (SCI-EXPANDED) database from Web of Science Core Collection as the literature source. Studies regarding cerebrovascular intervention were retrieved by the topic search, which included the title, abstract, and keywords of the articles. Search terms were acquired from Medical Subject Headings (MeSH).

We first searched the literature regarding cerebrovascular diseases. The core subject term “cerebrovascular disorders” and its subordinate subject terms which were “Basal Ganglia Cerebrovascular Disease,” “Brain Ischemia,” “Carotid Artery Diseases,” “Cerebral Small Vessel Diseases,” “Cerebrovascular Trauma,” “Intracranial Arterial Diseases,” “Intracranial Embolism and Thrombosis,” “Intracranial Hemorrhages,” “Leukomalacia, Periventricular,” “Sneddon Syndrome,” “Stroke,” “Vascular Headaches,” “Vasculitis, Central Nervous System,” and “Vasospasm, Intracranial” were searched. Besides, all the affiliate terms of the above subordinate subject terms such as “Intracranial Aneurysm” and” Brain Infarction” were included. We also searched for the relevant entry terms of all the subject terms. In the second part, researches related to interventional treatment were searched from the perspective of interventional radiography, interventional operation techniques, and interventional devices, with searched terms such as “interventional radiography,” “endovascular treatment,” and “stent,” respectively.

The intersection of the above two parts was included as research results. Papers only related to cardiac disease were eliminated by using exclusionary words such as “percutaneous coronary intervention” and “myocardial ischemia.” The literature type was restricted to “article.” Country/region was refined as “Peoples R China” or “Taiwan.” The year of publication was set from 1990 to 2019. Literature records were screened by 2 reviewers independently. The duplicated and unrelated articles were removed.

### Data collection and bibliometric analysis

The number of publications and bibliometric information of each article including citation times, years, authors, country and regions, journals, and keywords were extracted and recorded in Excel 2019. The quality and influence of publications were mainly measured by cited frequency and h-index which was a common evaluation index for literature. The citation report tool of Web of Science was used to analyze cited times and h-index among countries. Literature records were downloaded from Web of Science and imported into Citespace 5.6 software [10]. Network analysis and visualization were performed on Citespace by knowledge mapping. Cooperation network analysis was based on information of authors, institutions, and countries. The hotspots of the research field were reflected by the keywords co-occurrence network. The valuable articles, authors, and journals with high influence in the field were selected by co-citation analysis. We set the top 50 most cited or occurrence items each year as statistics selection criteria. Cluster visualization was generated to reflect the relationship between items. The emerging trend of hotspots was visualized by timeline view, in which the solid line indicated the persistence of new attention in this cluster. The fast increase in the co-occurrence of certain keywords and the burst in co-citation of specific articles over a period were identified by burstness detection. On the visualization figures, citation counts or occurred times were measured by the thickness of the “tree ring” with different colors. The width of color bands reflected the strength of the indicators at the year that the color represented. The relationship between key nodes was reflected in different lines. The color of the line indicated the year of first cooperation between two nodes and the thickness of the line represented the strength of their relationship. The lines with small nodes were partly omitted by the pathfinder algorithm for better visualization. Purple rings around nodes indicated high centrality. Cluster labels were extracted from keywords using latent semantic index (LSI) algorithm.

## Results

### Contributions in publications

**Fig. 1:**
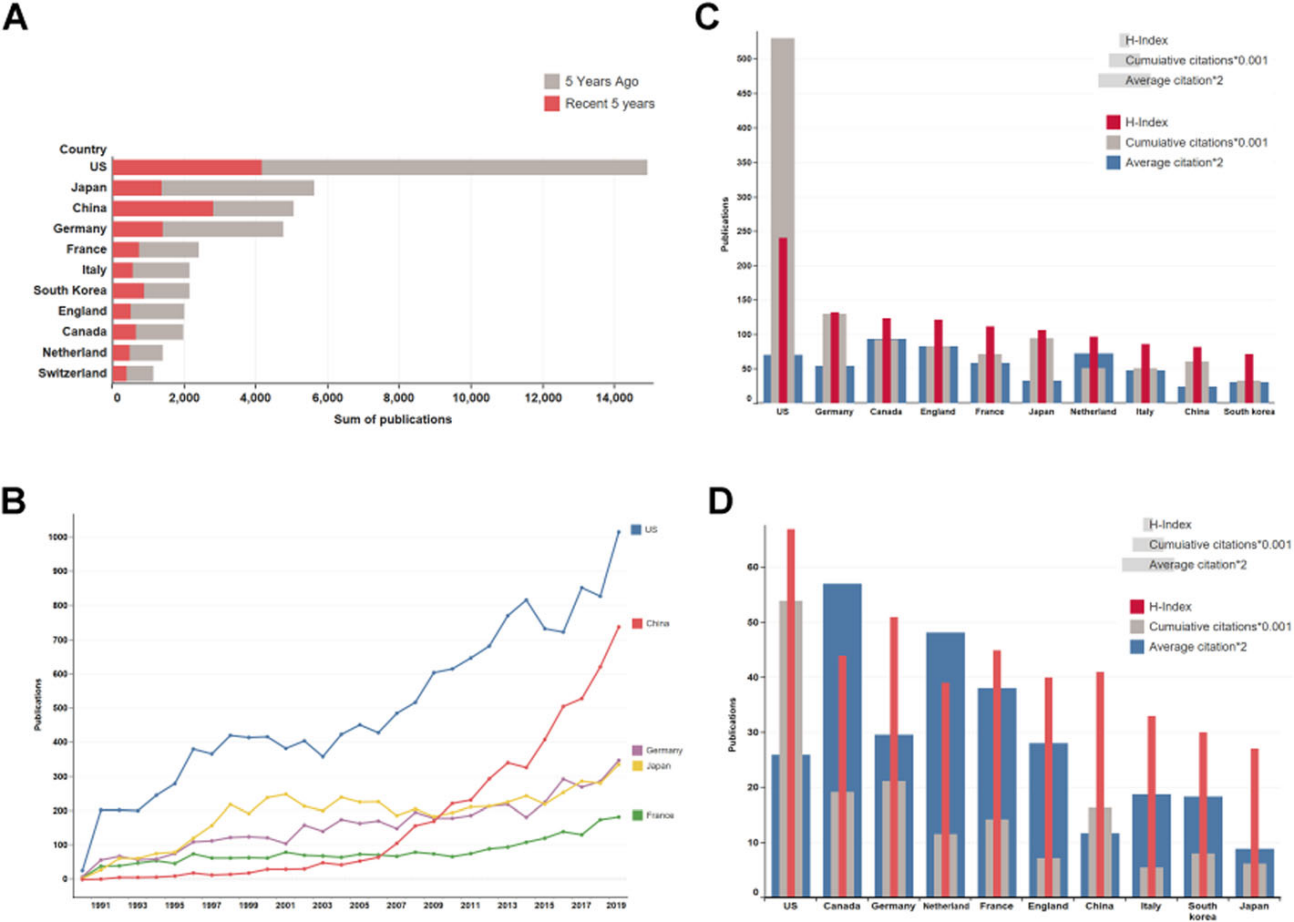
Publications in cerebrovascular intervention. **A** The top 10 countries that published most articles regarding cerebrovascular intervention. The red part represents the number of articles that were published in the last 5 years. China ranked third, and more than half of the articles were published in the last 5 years. **B** The trend in the number of publications of the five most published countries. Publications from China increased the fastest in recent years and reached the second place. **C** The quality of publications over the three decades in the 10 most published countries. The quality of the publications was evaluated by h-index, accumulated citations, and average citations. **D** The quality of publications in the past 5 years in the top 10 countries that published most articles

A total of 44,779 articles regarding cerebrovascular intervention from 1990 to 2019 were retrieved, in which 5052 publications were from Chinese researchers, including 2805 (55.5%) articles published in the last 5 years. The top five contributed countries to the global researches of cerebrovascular intervention were as follows: the USA (14,915), Japan (5,644), China (5,052), Germany (4,762), and France (2403) (Fig. 1A). Since 2010, the number of publications per year from China had come to second place. Furthermore, the average increasing rate of publications from China in the latest 5 years was 17.6%, which was much higher than that in the USA (6.8%), Japan (5.8%), Germany (7.4%), and France (7.8%) (Fig. 1B).

Publications from the USA had the most cumulative citation times (530,712), with average citation times of 35.58 and an h-index of 241. Germany came in the second place in terms of citation times and h-index, which were 129,613 times and 132, respectively. Canada, England, and the Netherlands were less in the number of publications but more in the number of high cited articles. The h-index of Canada, England, and the Netherlands were 124,121, and 97 respectively. There were 46.46 citations on average for each article published from Canada. This data ranked first among all countries, followed by England (41.16) and the Netherlands (36.26). In contrast, publications from China were cited 61,216 times, ranking seventh. The h-index of China was 82, ranking ninth. The number of publications in China was the third highest, but each article had only 12.12 citations on average (Fig. 1C). Considering the papers from China were mainly published within the latest 5 years (2805, 55.5%), the citation information from 2015 to 2019 was further analyzed. Although the average citations of each paper were only 5.44, the sum of citations (15,260 times) had come to the fourth place and the h-index was 39, ranking fifth in all countries. These improvements indicated a greater influence of China’s researches in the latest five years (Fig. 1D). To evaluate the articles with high influence, we identified 2006 articles, which has been cited more than 100 times, as high-influenced papers. Thereinto, 47 articles were involved with Chinese researchers, including 2 published in the 1990s, 28 published in the 2000s, and 17 published in 2010s. In the 30 articles published before 2010, only 8 (26.7%) articles’ first authors were from mainland China. Six (12.8%) were from Taiwan region, three (6.4%) were from Hong Kong, and thirteen (27.6%) were from other countries. For those published after 2010, the first authors in 10 of the 17 articles were Chinese researchers (9 were from mainland China). The increased proportion of the first author in these articles suggested that the contribution of China in high-quality articles was partly improved.

Articles from Chinese researchers were distributed in 631 journals. The journals that would like to publish articles from China were listed in Table 1. The top 10 journals published most articles from China were *World Neurosurgery* (IF=1.723, 229 articles), *Interventional Neuroradiology* (IF=1.450, 133 articles), *Stroke* (IF=6.046, 123 articles), *Chinese Medical Journal* (IF=1.555, 119 articles), *PLOS One* (IF=2.776, 114 articles), *American Journal of Neuroradiology* (IF=3.256, 114 articles), *Journal of Stroke & Cerebrovascular Diseases* (IF=1.646, 99 articles), *International Journal of Clinical and Experimental Medicine* (IF=0.181, 99 articles), *Medicine* (IF=1.87, 96 articles), and *Brain Research* (IF=2.929, 95 articles). These journals were included in 9 JCR categories.

**Table 1:** Ten journals with most publications from China

Journals	JCR category	Quartile in category	IF (2018)	Number of publications
*World neurosurgery*	Clinical neurology	Q4	1.732	229
	Surgery	Q3		
*Interventional neuroradiology*	Clinical neurology	Q4	1.45	133
	Radiology, nuclear medicine & medical imaging	Q4		
*Stroke*	Clinical neurology	Q1	6.046	123
	Peripheral vascular disease	Q1		
*Chinese medical journal*	Medicine, general & internal	Q3	1.555	119
*PLOS One*	Multidisciplinary sciences	Q2	2.776	114
*AJNR*	Clinical neurology	Q2	3.256	114
	Neuroimaging	Q2		
	Radiology, nuclear medicine & medical imaging	Q2		
*J stroke cerebrovasc*	Neurosciences	Q4	1.646	99
	Peripheral vascular disease	Q4		
*IJCEM*	Medicine, research & experimental	Q4	0.181	99
*Medicine*	Medicine, general & internal	Q2	1.87	96
*Brain research*	Neurosciences	Q2	2.929	95

### Cooperation network analysis focusing on China

**Fig. 2:**
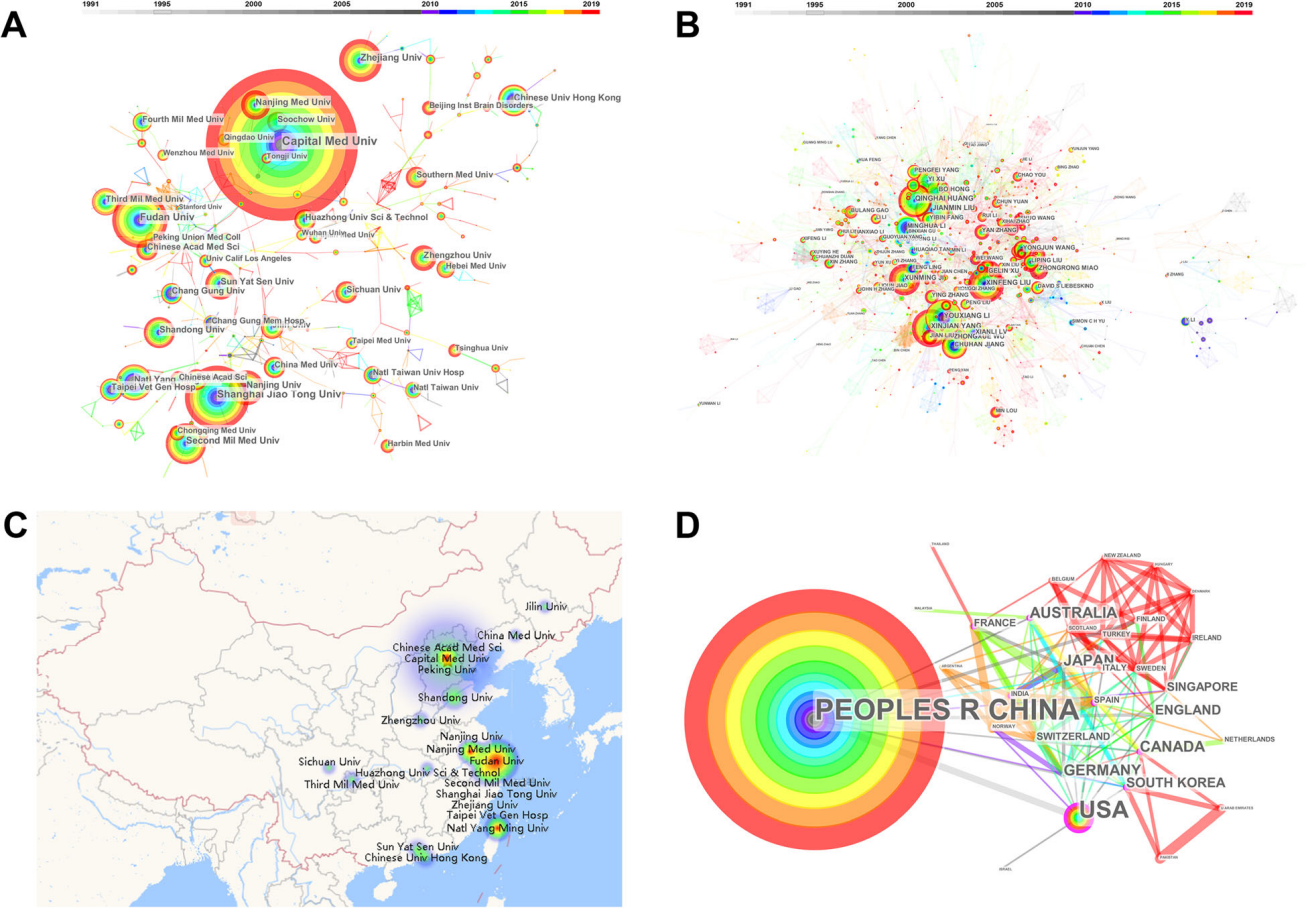
Cooperation network of the publications from China. **A** Institutions co-occurred network. The size of nodes represents the counts that institutions occurred in articles retrieved. The thickness of the lines represents the co-occurred counts of the connected two institutions. Time is reflected in different colors. **B** Authors co-occurred network. The size of nodes represents the counts that authors occurred in articles retrieved. The thickness of the lines represents the co-occurred counts of the connected two authors. Time is reflected in different colors. **C** The geological distribution of the ten most published institutions in China. Most of the institutions are located in east coast regions. **D** Countries co-occurred network. The size of nodes represents the counts that countries occurred in articles retrieved. The thickness of the lines represents the co-occurred counts of the connected two countries. Time is reflected by different colors

The cooperation network of institutions, authors, and countries based on the 5052 articles was visualized by knowledge mapping of Citespace 5.6 (Fig. 2A). Capital Medical Univ (685 articles, 13.56%) was the most prolific institution of China, followed by Shanghai Jiao Tong Univ. (276, 5.5%), Fudan Univ. (263, 5.2%), Second Mil Med Univ. (226, 4.5%), and Beijing Neurosurgical Institute (198, 3.92). The top 10 authors having published most articles were as follows: “XINJIAN YANG,” “YOUXIANG LI,” “XINFENG LIU,” “JIANMIN LIU,” “QINGHAI HUANG,” “XUNMING JI,” “BO HONG,” “ZHONGXUE WU,” “CHUHAN JIANG,” and “GELIN XU.” Interestingly, our result suggested that prolific authors were usually centralized in a few institutions. For instance, XINJIAN YANG and YOUXIANG LI were from Capital Medical Univ., XINFENG LIU and GELIN XU were from Nanjing General Hospital, and JIANMIN LIU and QINGHAI HUANG were from Second Mil Med Univ. (Fig. 2B). Most of these institutions were distributed in east coast regions, especially in Beijing and Shanghai, whereas the prevalence, incidence, and mortality of stroke in these regions were lower comparied with other provinces. The interventional treatment resource in China was unbalanced (Fig. 2C). As for the cooperation with other countries, the USA, Australia, Germany, Japan, and Canada were the five countries most frequently cooperated with China (Fig. 2D).

### Reference co-citation analysis

**Fig. 3:**
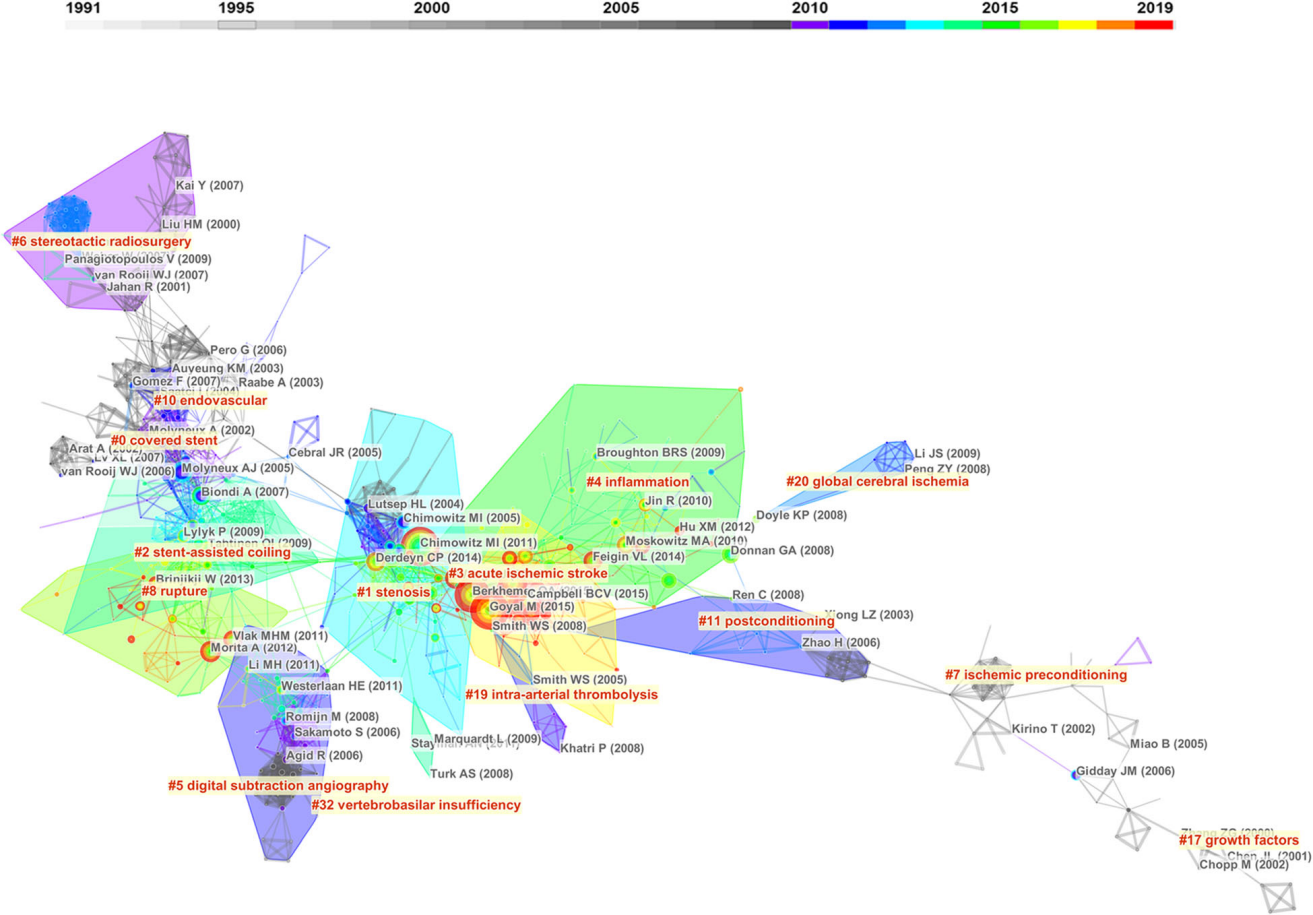
The network of the articles co-cited by retrieved articles from China. The size of the nodes represents the co-cited times of one article. The articles that areco-cited by the same article are connected with lines. The thickness of the lines reflects the co-cited counts. All the articles belonging to one cluster are covered by regions with different colors. Time is reflected by different colors

**Fig. 4:**
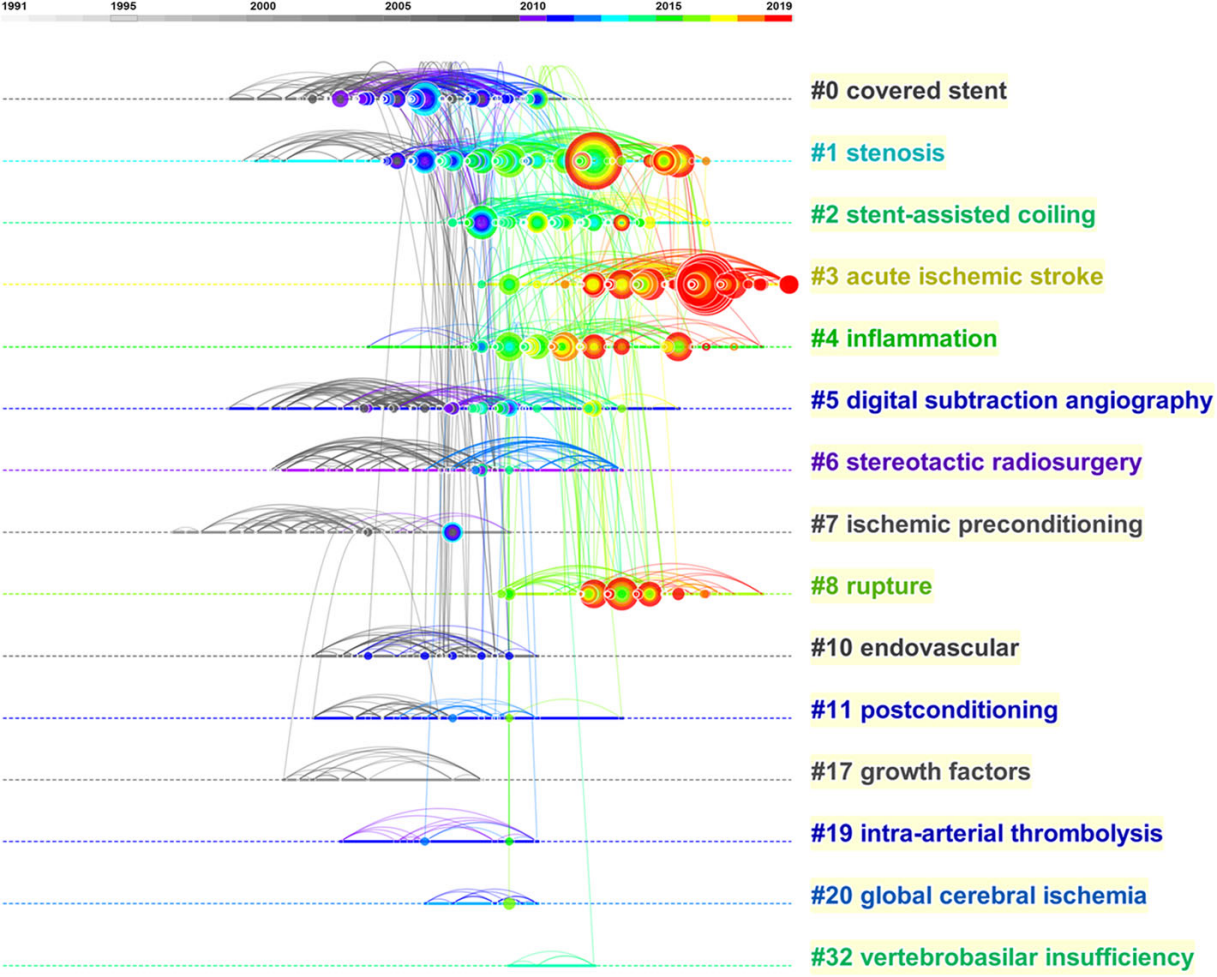
A timeline view of co-cited analysis. Papers with high cited counts are reflected by the nodes in the timeline axle of their clusters. Articles regarding acute ischemic stroke were most frequently co-cited by Chinese researchers in recent years

The articles cited by the included the 5052 articles were analyzed through co-citation analysis. The top 10 most co-cited articles were listed (Table 2). The top five articles were all randomized clinical trials regarding thrombectomy treatment of ischemic stroke in 2015, followed by one randomized clinical trial regarding stent treatment of carotid artery stenosis, two clinical guidelines or consensus, one meta-analysis about thrombectomy treatment, and one epidemiologic survey about Chinese intracranial atherosclerosis. Seven of the above 10 articles were published in the latest 5 years, indicating the burst of attention in cerebrovascular intervention researches. Six articles were concentrated on mechanical thrombectomy for ischemic stroke. On the visualization map, the co-cited articles were divided into 15 visualized clusters which were labeled by the LSI algorithm. These labels reflected the main topics of the cited literature included in one cluster. The top five clusters including the most co-cited articles were as follows: “covered stent,” “stenosis,” “stent-assisted coiling,” “acute ischemic stroke,” “inflammation,” and “digital subtraction angiography.” (Fig. 3). The timeline view suggested that the researches related to  “acute ischemic stroke” has been getting continuous attention since 2015. Studies involved with “inflammation” and “rupture” also attracted great attention in recent yearsy (Fig. 4).

**Table 2:** Ten articles most co-cited by the retrieved articles from China

Rank	Co-cited times	Year	Journal	Country	Title
1	154	2015	NEJM	Canada	Randomized assessment of rapid endovascular treatment of ischemic stroke.
2	146	2015	NEJM	Netherlands	A randomized trial of intraarterial treatment for acute ischemic stroke.
3	142	2015	NEJM	Australia	Endovascular therapy for ischemic stroke with perfusion-imaging selection.
4	133	2015	NEJM	USA	Stent-retriever thrombectomy after intravenous t-PA vs. t-PA alone in stroke.
5	131	2015	NEJM	USA	Thrombectomy within 8 hours after symptom onset in ischemic stroke.
6	81	2011	NEJM	USA	Stenting versus aggressive medical therapy for intracranial arterial stenosis.
7	67	2015	Stroke	USA	2015 American Heart Association/American Stroke Association Focused Update of the 2013 Guidelines for the Early Management of Patients with Acute Ischemic Stroke Regarding Endovascular Treatment
8	58	2013	Stroke	USA	Recommendations on angiographic revascularization grading standards for acute ischemic stroke: a consensus statement.
9	58	2016	Lancet	Canada	Endovascular thrombectomy after large-vessel ischaemic stroke: a meta-analysis of individual patient data from five randomised trials.
10	49	2014	STROKE	China	Prevalence and outcomes of symptomatic intracranial large artery stenoses and occlusions in China: the Chinese Intracranial Atherosclerosis (CICAS) Study.

*NEJM The New England Journal of Medicine*

### Keywords co-occurrence analysis

**Fig. 5:**
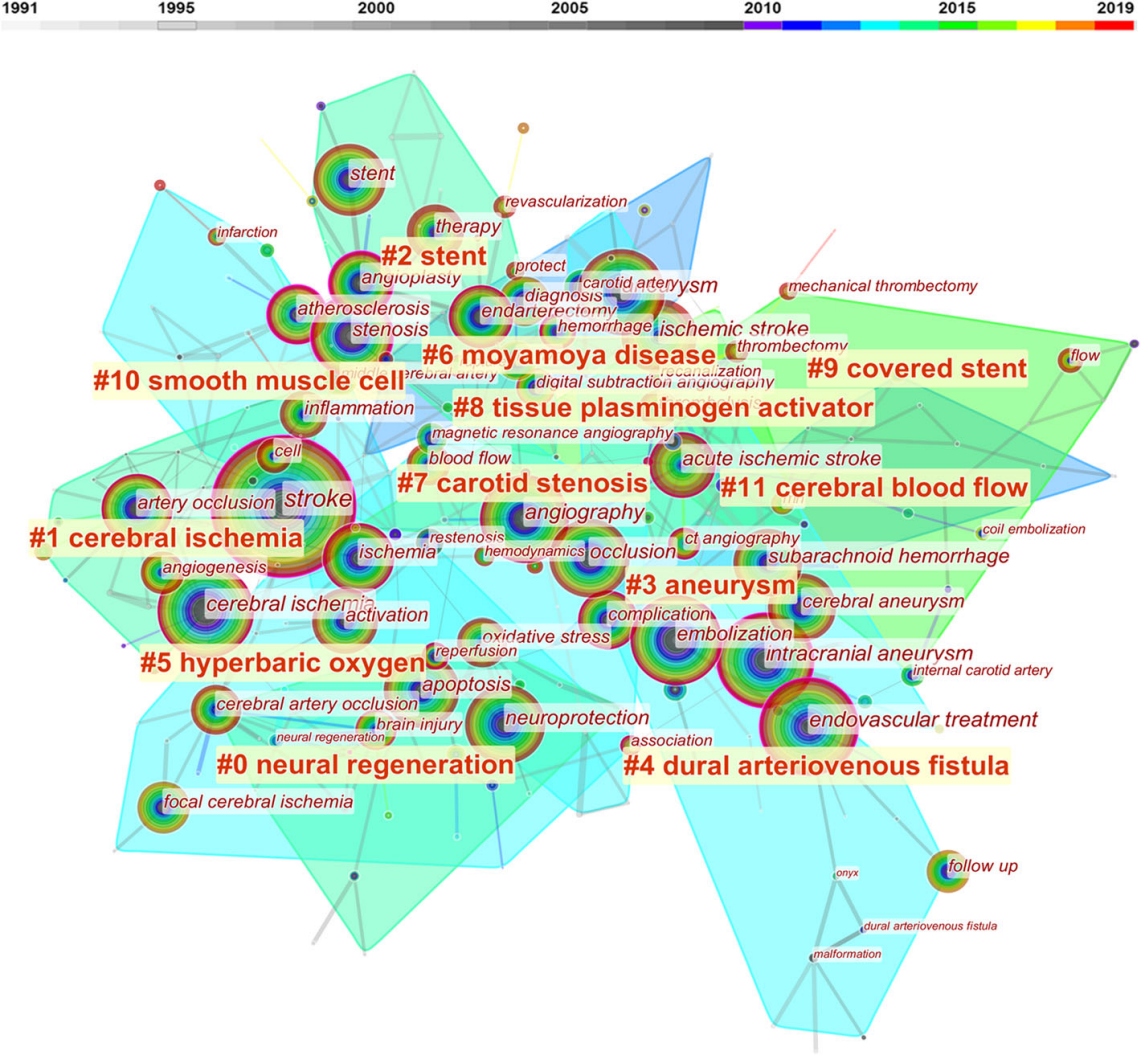
Co-occurrence network of keywords. The size of the nodes represents the co-occurred counts of keywords. The keywords that are co-cited by the same article are connected with lines. All the keywords belonging to one cluster are covered by regions with different colors. Time is reflected in different colors

**Fig. 6:**
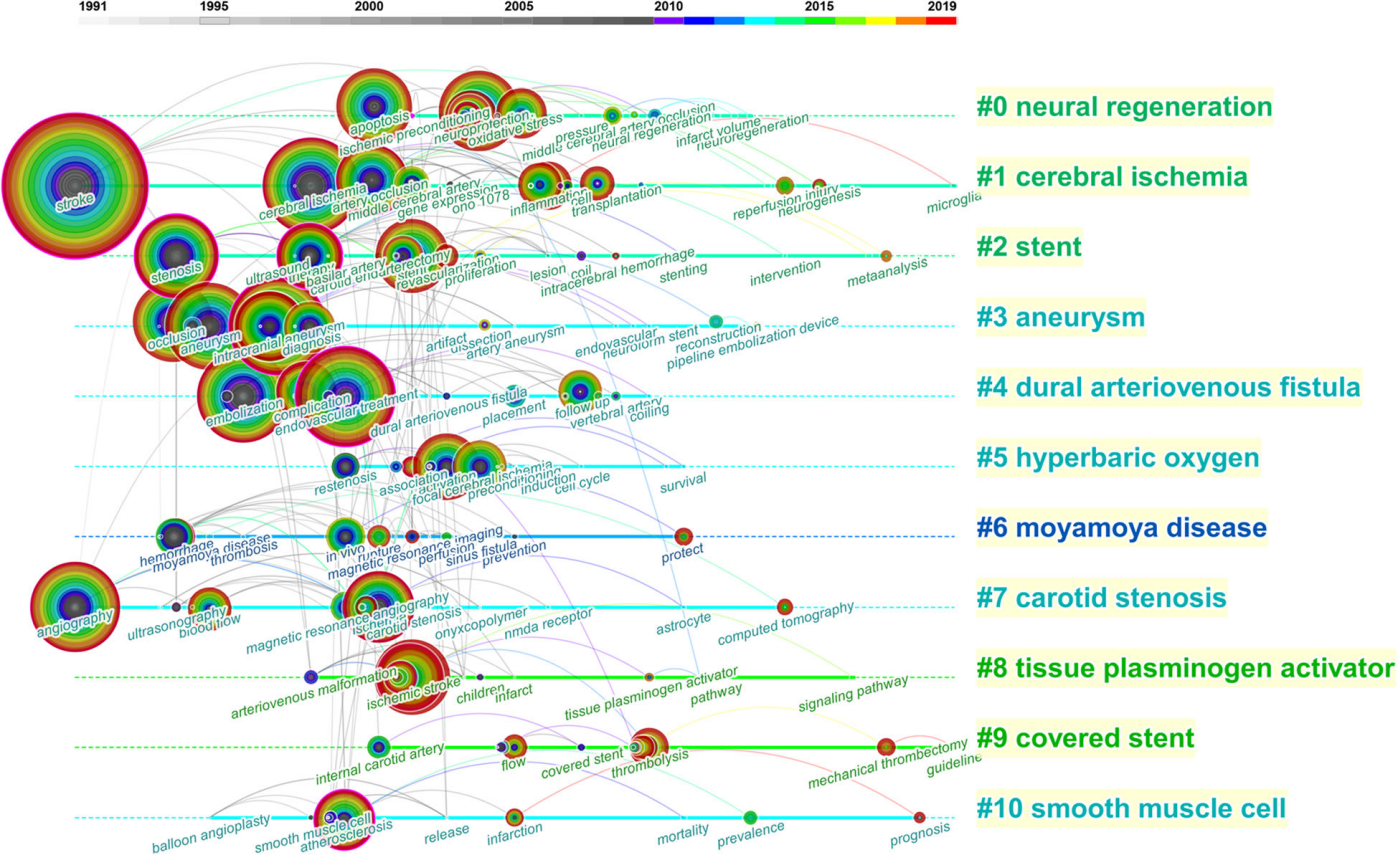
Timeline view of keywords analysis. Hot spots of one cluster were reflected by the nodes in the timeline. Clusters with more large nodes reflected the hot field of cerebrovascular intervention

The hot spots of cerebrovascular intervention in China were detected by the knowledge mapping of keywords. Fifty most occurred keywords each year in the past three decades were selected to construct the keywords co-occurrence network. “Stroke,” “endovascular treatment,” “intracranial aneurysm,” “ischemic stroke,” “cerebral ischemia,” “ischemic stroke,” “cerebral ischemia,” “aneurysm,” “angiography,” and “neuroprotection” were 10 keywords that occurred mostly in the network. Eleven clusters were identified by the “found cluster” function of Citespace 5.6 and labeled by the LSI algorithm (Fig. 5). The cluster label names ordered as the number of keywords in the clusters were as follows: “neural regeneration,” “cerebral ischemia,” “stent,” “aneurysm,” “dural arteriovenous fistula,” “hyperbaric oxygen,” “moyamoya disease,” “carotid stenosis,” “tissue plasminogen activator,” “covered stent,” “smooth muscle cell,” and “cerebral blood flow.” Five names of diseases were selected as labels. All the articles regarding these diseases were retrieved from our above search results. We found 2525 (50.0%) articles related to cerebral ischemia, 1255 (24.8%) for intracranial aneurysm, 105 (2.1%) for dural arteriovenous fistula, 131 (2.6%) for moyamoya disease, and 584 (11.6%) for carotid stenosis. The timeline view showed that “cerebral ischemia” was always a research hotspot since 1991 until now, and “covered stent” was another continuous research hotspot since the 2000s (Fig. 6). Five keywords with the strongest citation burst were “carotid artery,” “restenosis,” “nitric oxide,” “MR angiography,” and “arteriovenous malformation” (Table 3).

**Table 3:** Top 20 Keywords with the Strongest Citation Bursts

Keywords	First occurred	Strength	Burst begin	End
Carotid artery	1991	17.5523	1996	2011
Restenosis	1991	15.7391	2001	2012
Nitric oxide	1991	13.4386	2001	2011
MR angiography	1991	11.8505	1997	2009
Arteriovenous malformation	1991	11.802	2000	2012
Malformation	1991	10.2963	1998	2009
Guglielmi detachable coil	1991	9.9596	2001	2010
Ultrasonography	1991	9.6952	1996	2009
Dural arteriovenous fistula	1991	8.8745	2004	2011
Gene expression	1991	6.2523	2004	2009
Embolization	1991	6.1641	1998	2001
Smooth muscle cell	1991	5.9877	2000	2009
Thrombosis	1991	5.7673	1998	2008
Angiography	1991	5.3289	1993	1999
Transcranial Doppler	1991	5.021	2003	2009
CT	1991	4.9913	2003	2007
Cerebral infarction	1991	4.7254	2003	2008
Artery stenosis	1991	4.6447	1996	2003
Diagnosis	1991	4.2921	2003	2007
Ischemic preconditioning	1991	3.4546	2003	2006

## Discussion

Our bibliometric study analyzed the contribution of each country to the development of cerebrovascular interventional research, especially the contribution of China. The contributions and cooperation of Chinese institutions and authors were evaluated and ranked. The co-citation analysis presented valuable researches in this field and primarily showed promising research directions. The co-occurrence analysis of keywords further pointed out the research hotspots and the future research directions in this field.

### Current situation of Chinese cerebrovascular interventional researches

China is one of the largest developing countries, where stroke is the leading cause of death and disability, with 11 million cases of stroke annually [11, 12]. A study indicated that China was a country with a high stroke burden but little research regarding cerebrovascular disease [13]. However, in the latest 5 years, the number of publications related to cerebrovascular intervention in China grew most rapidly. This might attribute to the reforms of China’s healthcare system which enabled more patients get access to this new technique and extended the sources of clinical cases [14]. Since the establishment of the Ministry of Health China Stroke Prevention Project Committee in April 2011, more stroke centers in the nation were constructed y and more people could accept endovascular treatment immediately after the onset of stroke[6]. Nevertheless, the sum of publications citations, average publication citations, and h-index were all still low in the ranking, despite the condition was improving in the last 5 years. In the 2,006 articles cited more than 100 times, 47 articles were involved with Chinese researchers, which merely accounted for 2.3%. Very few journals in Q1 (First quartile in JCR Category) were in the list of journals that Chinese researchers most published. These data denoted that cerebrovascular interventional researches in China have reached a considerable scale, but the quality needs further improvement.

The contributions of researchers and medical institutions of China are critical for the blossom of cerebrovascular interventional researches. In the 1990s, Wu ZX, Ling F, etc. began to report their early experience of treating intracranial aneurysms, traumatic carotid cavernous fistulas, and arteriovenous malformation with endovascular techniques [15–17]. As the interventional techniques and materials in China was deficient, they produced self-made tungsten coils to treat aneurysms and achieved favorable outcomes [18]. The first cerebrovascular intervention training institution was established in 1996, which attracted many Chinese doctors to learn and popularize endovascular treatment nationally. Cooperation network analysis suggested that institutions including Capital Medical Univ., Shanghai Jiao Tong Univ., Second Mil Med Univ., and their affiliated hospitals have made a significant contribution to the cerebrovascular interventional research of China. Yang XJ and Li YX first reported the application of Neuroform stent in intracranial aneurysm in domestic patients [19]. Liu JM et al. first reported stent-assisted electrical detachable coil embolization in vertebral artery aneurysms and intracranial ruptured aneurysms in China [20]. In 2004, embolization with onyx in  arteriovenous fistula and malformation was widely applied [21]. The primary experience of Wingspan stent in Chinese patients was reported by Fan XY, Liu XF, etc. [22]. However, most of these studies were small and reported on domestic journals in China. Only a few articles were published in international journals, which limited their global influence. Recently, with the improvement of the medical environment and the increasing health burden on Chinese patients, Chinese cerebrovascular intervention developed rapidly. More original researches were designed and successfully answered focus issues in this field. Results of the DRICT-MT trial first indicated endovascular thrombectomy with or without intravenous alteplase had similar outcomes in large vessel occlusion induced ischemic stroke, within a 20% margin of confidence [23]. The BASILAR trial proved that in patients with acute basilar artery occlusion, endovascular treatment administered within 24 h was associated with better functional outcomes and reduced mortality than standard medical treatment alone [24]. The BEST trial showed no evident favorable outcomes of patients receiving endovascular therapy compared with those receiving standard medical therapy alone in vertebrobasilar artery occlusion [25]. These trials represented a growing influence of China on the global cerebrovascular interventional researches.

Cooperation networks of institutions and authors inferred that most of the authors with high citations were from a few institutions that were distributed in east coast regions, especially in Beijing and Shanghai [26]. This phenomenon reflected the reality of the imbalanced distribution of medical resources. Further investment to help unbalanced regions is needed [11]. The publication of high-qualified clinical trials on top-tier journals indicated that multi-center researches in China were feasible and promising. Therefore, enhancing inter-institutional cooperation may be a cost-effective way to improve the general research level of China.

### The research trend of cerebrovascular intervention in China

The result of reference co-citation analysis indicated a burst of attention in cerebrovascular intervention researches in the last 5 years. Chinese researchers paid much attention to thrombectomy treatment of ischemic stroke, which suggested this field has great potential. From the most occurred 10 keywords, we can find that “intracranial aneurysm” and “cerebral ischemia” were two important topics in cerebrovascular intervention. In the keyword analysis, all the keywords were divided into 11 clusters, which represented different research directions. The clusters were ranked by the number of keywords in each cluster. Cluster #0 labeled by “neural regeneration” was the one comprised most keywords. The highly occurred keywords in this cluster, such as “neuroprotection,” “apoptosis,” and “oxidative stress” were mainly involved in basic experimental researches, which denoted the close relationship between clinical and basic researches in the cerebrovascular intervention field. One of the special contributions of China was the exploration of the neuroprotective effect of traditional Chinese medicine, such as Sanhua Decoction and QiShenYiQi [27, 28]. Many trials about neuroprotection failed to translate their protective effects from animal models to humans. We could see that the solid line of cluster#0 halted in recent years on the timeline view. Still, approximately half of the patients who suffered from ischemic stroke do not regain independent function after successful endovascular recanalization, which is a strong impetus for further investigation on neuroprotection [29]. Cluster#1 was labeled “cerebral ischemia.” Timeline span suggested this was the only cluster with continuous attention throughout the three decades. In this cluster, new key nodes with a tendency to enlarge such as “stroke,” “artery occlusion,” and “reperfusion injury” continuously emerged on the timeline. According to the result of the co-citation mentioned above, cerebral ischemia would still be a substantial research trend in the future. Cluster#2 and #9 were labeled by “stent” and “covered-stent,” respectively, suggesting the importance of new stent in interventional techniques. “Stenosis” and “carotid endarterectomy” were two keywords with large nodes in the network, which might due to the long-time discussion about the strategy selection of stenting or endarterectomy in different cases. The time span of the cluster#2 and #9 extended to 2017 or later, indicating persistent attention of stent treatment. The wide application of different stents, including stent-assisted embolization, stent-assisted angioplasty, and the outspring of novel stents such as flow diverters and stent retrievers, made stents popular for endovascular treatment. Cluster#3 was labeled “aneurysm.” Key nodes in this cluster, such as “intracranial aneurysm,” “cerebral aneurysm,” and “subarachnoid hemorrhage,” were all intensely related to the management of aneurysm. The timeline view showed that these nodes intensely emerged in the 1990s, denoting that traditional interventional therapies such as coil embolization had been introduced and widely studied in China then. Later, researchers realized that stents with high material coverage could become a stand-alone device to embolize aneurysms by changing the hemodynamics. Chinese researchers invented the Tubridge flow diverter and demonstrated a significantly higher obliteration rate of large and giant aneurysms during a 6-month follow-up with this device than stent-assisted coiling [30]. Flow diverter treatment for aneurysms became a hotspot in recent years. Timeline span suggested topics in cluster#4, cluster#5, and cluster#6 were not the mainstay in recent years. Endovascular embolization had become a first-line treatment for dural arteriovenous fistula, direct carotid cavernous fistula, and arteriovenous malformation. The cure rate with Onyx was considerably acceptable [31]. Breakthrough progress in hyperbaric oxygen and clinical management for moyamoya disease was not significant. Key nodes in cluster#7 “carotid stenosis” suggested that interventional radiology, such as “angiography” and “magnetic resonance angiography,” were often co-occurred with carotid stenosis, indicating the frequent application of interventional techniques in carotid stenosis.

### Limitations

Our study has limitations. First, we chose the SCI-EXPANDED database as the only source for English literature retrieval owing to the data format for analysis, so the comprehensiveness of literature retrieval may be limited. Second, in the judgment of articles from China, we enrolled all the eligible articles that were involved with Chinese researchers, including a fraction of studies initiated by authors from other countries. Our researches may overestimate the contributions of Chinese researchers. Third, our study did not enroll papers during the COVID-19, which have a profound impact on global medical researches and brought changes to cerebrovascular studies. Further investigation is valuable for this special period.

## Conclusions

Researches for cerebrovascular intervention increase rapidly in China over the decades, but the quality of research still needs further enhancement. Most of these researches concentrated on diseases with high incidences in China, such as cerebral ischemia and intracranial aneurysm. With the fast development of regional stroke centers, more Chinese researchers will join in and contribute to the questions of cerebrovascular intervention with global concern.

## Data Availability

The datasets used and analyzed during the current study are available from the corresponding author on reasonable request.

## Abbreviations

CVDs: Cerebrovascular diseases
LMICs: Low-income and middle-income countries
MeSH: Medical Subject Headings
